# Identification of Implementation Strategies Used for the Circle of Security-Virginia Family Model Intervention: Concept Mapping Study

**DOI:** 10.2196/10312

**Published:** 2018-06-14

**Authors:** Bettina Nielsen, Kari Slinning, Hanne Weie Oddli, Filip Drozd

**Affiliations:** ^1^ Department of Psychology University of Oslo Oslo Norway; ^2^ Network for Infant Mental Health Regional Center for Child and Adolescent Mental Health, Eastern and Southern Norway Oslo Norway

**Keywords:** implementation strategies, Circle of Security Virginia-Family Model, Expert Recommendations for Implementing Change taxonomy, methodology, reproducibility, knowledge transfer

## Abstract

**Background:**

A reoccurring finding from health and clinical services is the failure to implement theory and research into practice and policy in appropriate and efficient ways, which is why it is essential to develop and identify implementation strategies, as they constitute the *how-to* component of translating and changing health practices.

**Objective:**

The aim of this study was to provide a systematic and comprehensive review of the implementation strategies that have been applied for the Circle of Security-Virginia Family (COS-VF) model by developing an implementation protocol.

**Methods:**

First, informal interviews and documents were analyzed using concept mapping to identify implementation strategies. All documentation from the Network for Infant Mental Health’s work with COS-VF was made available and included for analysis, and the participants were interviewed to validate the findings and add information not present in the archives. To avoid lack of clarity, an existing taxonomy of implementation strategies, the Expert Recommendations for Implementing Change, was used to conceptualize (ie, name and define) strategies. Second, the identified strategies were specified according to Proctor and colleagues’ recommendations for reporting in terms of seven dimensions: actor, the action, action targets, temporality, dose, implementation outcomes, and theoretical justification. This ensures a full description of the implementation strategies and how these should be used in practice.

**Results:**

Ten implementation strategies were identified: (1) develop educational materials, (2) conduct ongoing training, (3) audit and feedback, (4) make training dynamic, (5) distribute educational materials, (6) mandate change, (7) obtain formal commitments, (8) centralize technical assistance, (9) create or change credentialing and licensure standards, and (10) organize clinician implementation team meetings.

**Conclusions:**

This protocol provides a systematic and comprehensive overview of the implementation of the COS-VF in health services. It constitutes a blueprint for the implementation of COS-VF that supports the interpretation of subsequent evaluation studies, facilitates knowledge transfer and reproducibility of research results in practice, and eases the replication and comparison of implementation strategies in COS-VF and other interventions.

## Introduction

### Background

Using interventions that aim to change health provider behavior can be an effective way of improving health outcomes and reducing health costs [1]. At the same time, one of the most consistent findings from research on health and clinical services is the failure to implement theory and research into practice and policy [2] and sustain the use of interventions and their effects in practice [3]. Therefore, several researchers argue that there is an urgent need for methods of specifying and reporting interventions in ways that strengthen the knowledge base necessary to enable interventions to be more effective, replicable, and implementable (see eg, [4-6]).

There are several barriers that hamper successful implementation of innovations. A lack of conceptual clarity, for example, has made it difficult to identify, develop, and test implementation strategies. First, the terms and definitions for implementation strategies are used inconsistently. Second, the description for implementation strategies are not detailed enough to enable scientific or real-world replication, which is one of the basic premises of research [7]. This has until recently, partly been because of a lack of reporting guidelines for implementation studies and strategies (for recent standards, see [8]). Thus, essentially, the way in which intervention research is reported, generally fails to contribute toward a cumulative science of interventions. Other problems that occur is that implementation strategies are rarely justified theoretically, they either lack operational definitions or manuals to guide their use, or are part of a multifaceted, packaged approach whose specific elements are poorly understood [5]. It also obscures the interpretation and understanding of outcomes in intervention studies. On the one hand, with ineffective interventions, it becomes practically impossible to know whether it was the intervention itself that failed, the way it was integrated in practice, or both. On the other hand, it is difficult to understand how to integrate and embed a presumably effective intervention into practice without systematic and comprehensive protocols for their implementation.

The significant gap between what we know and what we do (ie, the “how-to” when translating research findings into daily practice) challenges effective and efficient health care services [9], which is why much of the scientific literature emphasizes the need to understand the barriers to delivering optimal health care and applying research into practice. To bridge this gap, it is our belief that implementation protocols should be routinely published for all interventions, in a similar way as study and intervention protocols. This will contribute to accumulate and extend the evidence-base for intervention and implementation research and improve future decisions regarding the implementation of interventions among policy and decision makers, health services, practitioners, and other stakeholders (eg, determining whether implementing an intervention into existing practice is feasible and acceptable). Furthermore, as research resources are finite, implementation protocols will also help the scientific community avoid unnecessary and duplicate research because of inconsistencies in language or inadequate descriptions. By clearly specifying and reporting strategies used to embed an intervention into practice, it will also ease the interpretation of research findings and contribute to research syntheses (eg, systematic reviews and meta-analyses).

### Circle of Security-Virginia Family Model

The Circle of Security-Virginia Family (COS-VF) model is an intervention developed for primary caregivers (eg, biological parents, foster parents, and adoptive parents) with children who have or are at risk of developing attachment problems [10]. COS-VF is designed to intervene in areas related to caregiver-child relationships; attachment, exploration, behavior management, and emotion-regulation. The core constructs involve Bowlby [11] and Ainsworth’s [12] ideas of a Secure Base and Safe Haven, and the purpose is to convey these ideas to caregivers in a way that is tangible, as well as easy to practice in their daily life. The treatment follows a manual that is divided into six different phases: (1) families are assessed and a treatment plan is prepared, (2) establishing a safe base when working toward change, (3) learning the COS-VF framework, (4) developing observation abilities, (5) increasing the caregivers reflective functioning, and (6) empathic shift, assessment of change, and end of treatment. Each phase has different goals for learning, and the therapists evaluate how long it takes to acquire learning goals in the different phases for each individual family. It is often 20 to 30 hours before the entire manual has been reviewed and the change targets have been reached.

**Figure 1:**
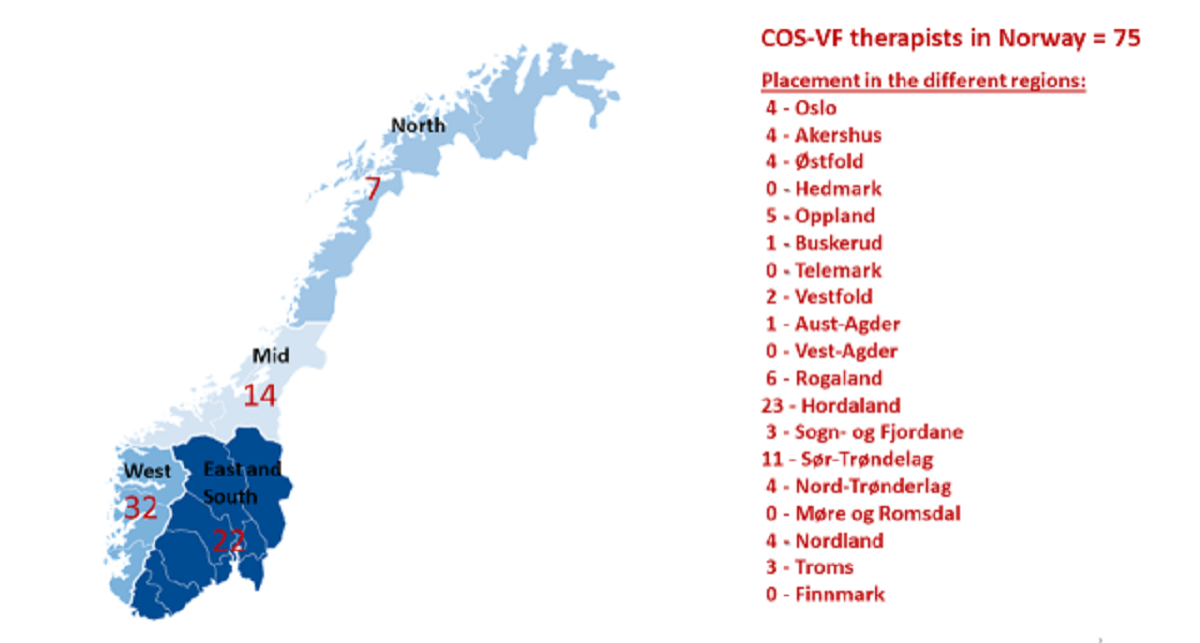
The current state of the implementation of Circle of Security-Virginia Family (COS-VF) model in Norway.

The Network for Infant Mental Health (NIMH) in Norway, which is responsible for training and implementation of interventions in the field of infant mental health, established a collaboration with Robert Marvin and William Whelan (ie, COS-VF developers) in 2009 to learn the COS-VF intervention. The goal was to gradually take complete responsibility for the COS-VF training and supervision in Norway. This was a stepwise educational process where a group of clinical psychologists at NIMH, first, became certified in using the Secure Base-Safe Haven coding system (SBSH-CS; Marvin and Whelan, unpublished data, 2007 [13]) for the Strange Situation Procedure (SSP, [12]), then as COS-VF therapists, then as COS-VF supervisors and, finally, as teachers for future COS-VF therapists. The current state of the implementation in Norway is depicted in Figure 1.

Clinical manuals and instructions on how to use the materials in the intervention were provided, but there were no manuals or instructions on how to implement the intervention in an effective way in clinical settings. The COS-VF providers at NIMH used the core implementation components proposed by Fixsen and Blasé [14] (ie, recruitment and selection, pre- and in-service training, consultation and coaching, staff evaluation, decision support data systems, facilitative administrative supports, and systems interventions) informally to implement COS-VF in the best possible way. This is because core implementation components are “by definition, essential to achieving good outcomes for those targeted by the intervention” [15]. However, no formal implementation plan was ever designed (ie, much remained tacit knowledge), and thus far, there is still no formal implementation protocol that can facilitate the implementation process for either COS-VF providers or COS-VF therapists in Norway or internationally.

To our knowledge, there is no published research on COS-VF to date. However, the core components in COS-VF are similar to the content of the original 20-week, group-based COS protocol that has shown promising results in quasi-experimental studies (see, eg, [16-18]), with the more recent addition of a small randomized trial that demonstrated its effectiveness on preschool children’s attachment and well-being [19]. The main differences between the intervention protocols are its group-based format and focus on caregivers’ core sensitivities, whereas COS-VF therapists work with individual families, focusing on strategies used to navigate close relationships and protect caregivers from emotional distress. Moreover, there are several other versions of COS (eg, COS-Parenting and the COS Virginia-Group model), and training in these interventions is offered both in Norway and internationally, which is yet another reason there is a need for an implementation protocol that clearly describes the different ways in which these are implemented in health and clinical services. The aim of the study was, therefore, to provide a systematic and comprehensive review of the implementation strategies that have been applied for COS-VF by developing an implementation protocol.

## Methods

### Protocol Development

This study is part of a larger project investigating the implementation of COS-VF in Norway. The first step in this process was to develop an implementation protocol for the intervention, which commenced by identifying implementation strategies, defined as “methods or techniques used to enhance the adaption, implementation and sustainability of a clinical program or practice” [5], but more easily understood as the *how-to* component of innovation in health care. The second step was to use Proctor and colleagues’ [5] recommendations for reporting, which are the fundamental principles of *naming*, *defining*, and *specifying or organizing* implementation strategies (also see Analysis below).

### Participants

The main work and data in this study consisted of analyzing all the documents in the COS-VF archives. Moreover, to validate the findings, we (ie, BN and FD) conducted interviews with two of the key personnel involved in the implementation of COS-VF; ie, one COS-VF supervisor and one staff member at the NIMH, who provide technical and administrative support to COS-VF therapists. The number of COS-VF supervisors and administrative staff is very limited; only six supervisors and one staff member are involved with technical and administrative support. However, a very knowledgeable supervisor was interviewed. The supervisor lived in the same city as the researchers (which facilitated ongoing contact and meetings) and was one of the first in Norway to become certified as a COS-VF therapist and supervisor, and thus, has extensive experience with the intervention.

### Procedure

At first, the researchers (BN and FD) were provided with access to the archives at NIMH that contain all the information and documentation concerning COS-VF. In parallel, informal interviews were used to help identify key implementation strategies in COS-VF. Informal interviews do not use a prepared set of interview questions, rather they have a repertoire of questions they draw upon when appropriate [20]. In this study, the repertoire was theory-driven, based on the core implementation components proposed by Fixsen and colleagues [21], although the analysis was conducted independent from their model. While working with identifying strategies and describing them, the researchers (BN and FD) were in ongoing contact with the participants to acquire further information when needed or revise the preliminary mapping of implementation strategies. Finally, participants were asked to review the final results of the analysis to make sure all central strategies were identified and correctly described.

### Analysis

All documents concerning COS-VF in the NIMH database were analyzed by means of concept mapping, to identify a distinct set of implementation strategies and their interrelationships. In this study, conceptual mapping refers to a specific integrated approach of concept mapping, described as “a structured methodology for organizing the ideas of a group or organization, to bring together diverse groups of stakeholders and help them rapidly form a common framework that can be used for planning, evaluation, or both” [22]. This approach facilitates collection of information from different participants and other data sources in practically any scenario in which an issue or need requires definition, planning, and evaluation and enables feedback on these data to participants in a timely manner.

First, all of the documents or materials in the archives were carefully reviewed and manually organized based on whether they were relevant to implementation or not. There were 104 items (ie, emails, Word or PDF documents, video materials, and pictures) in the archives, only 21 one of which were used in the analysis. These items included (1) NIMH’s emails sent to the students about the educational program and the NIMH’s cloud service (will be further explained in the Results section), (2) descriptions of the educational program, (3) educational certificates for therapists and supervisors, (4) descriptions of the core sensitivities revised and adjusted for Norwegian conditions, (5) instructions for the SSP, (6) transcription templates (for transcribing SSP), (7) overview of videos to use during lectures and for certification, (8) confidentiality agreements (all of the mentioned items were developed by the NIMH), (9) the COS interview (developed by Bert Powell et al), (10) the SBSH-CS (developed by Bob Marvin and William Whelan), and (11) the certification criteria (developed by Bob Marvin, William Whelan, and the NIMH). BN and FD also got access to the NIMH’s cloud service where there were 20 SSP training videos (ie, of families in the SSP) and given the printed COS-VF manual (developed by Bob Marvin and William Whelan; translated into Norwegian by NIMH). The other items in the archives were excluded because they were either irrelevant for the implementation, old versions (new ones were available), or duplicates. None of the items used for the implementation were peer-reviewed.

As we were following Proctor and colleagues’ [5] guidelines for specifying and reporting implementation strategies, the next step was to identify and name the strategies involved in the COS-VF implementation. *Naming* refers to a process of labeling a strategy, preferably using language that is consistent with existing literature. To support this process, we applied the Expert Recommendations for Implementing Change (ERIC­: [7]) taxonomy to identify and name the unique implementation strategies. Each of the documents or materials in the archives was organized based on where they belonged within the different strategies in the ERIC taxonomy. After all of the documents or materials had been categorized, the participants were interviewed to verify that the different documents or materials BN had categorized and described were done so correctly, and furthermore, to give additional information about the implementation process that could not be identified by going through the archives. After the findings had been validated and the different strategies were identified, the next step in Proctor and colleagues’ [5] guidelines was to define and organize the implementation strategies. *Defining* is conceptually describing what the strategy involves, whereas *organizing* entails operationalization of the core strategies according to seven dimensions: (1) the actors (ie, who delivers the strategy), (2) the actions (ie, what will be done), (3) action targets (ie, toward what or whom and at what level), (4) temporality (ie, when or in what phase), (5) dose (ie, at what frequency and intensity), (6) implementation outcome(s) affected, and (7) justification (ie, theoretical, empirical, or pragmatic justification).

Proctor and colleagues’ [23] taxonomy of implementation outcomes was used to label implementation outcomes (ie, dimension 6) to make sure there was clarity concerning the terms used to describe these. This taxonomy consists of the following implementation outcomes: (1) acceptability (ie., the belief that the innovation is agreeable, palatable, or satisfactory), (b) adoption (ie, the decision, intention, or action to try and employ the innovation), (c) appropriateness (ie, the perceived suitability, applicability, or compatibility of the innovation), (d) feasibility (ie, the extent to which the innovation can be carried out), (e) implementation cost (ie, the cost impact of the implementation effort), (f) penetration (ie, the way a practice is integrated within service settings and its subsystems), and (g) sustainability (ie, the way the innovation is maintained within the organizations ongoing operations).

A concept map was designed to conceptualize how the different strategies were related to each other. As a last step, the implementation strategies were organized within Fixsen and Blasé’s [14] diagram of core implementation components, as they were the inspiration for the NIHM’s implementation process. The participants were given access to the result section to validate and give feedback on all of the results before the implementation protocol was submitted for publication.

### Post-Hoc Application

Ideally, an implementation protocol should be developed during the planning stage of an intervention; however, it can also be developed in a reflective or evaluation phase, as in this study, which is an important part of the implementation. Several implementation theories such as Fixsen’s [24] Active Implementation Framework (AIF), the Dynamic Adaptation Process model [25], and Consolidated Framework for Implementation Research model [26] acknowledge the importance of evaluation and reflective phases in continuous cycles of quality improvement. Implementation theories such as the AIF framework were used informally to plan the implementation of COS-VF, thereby making a post-hoc implementation protocol both feasible and informative. A systematic and comprehensive implementation protocol can help identify, synthesize, and critically appraise the implementation of an intervention and help prevent “type III” errors (ie, correctly rejecting the effectiveness of an intervention when the intervention was inadequately implemented or delivered; [27]). Thus, it is better to develop an implementation protocol post-hoc, rather than never. This may contribute to identifying potential improvements to the continued implementation of COS-VF (or other interventions) and understanding of subsequent research results. More generally, it may also contribute to documenting practical implementation of interventions in health care services and provide “lessons learned” for researchers, clinicians, and decision makers (eg, when to use the same implementation strategies or devise new strategies), especially when viewed conjointly with results from evaluation studies.

## Results

### Implementation Strategies

After analyzing all the documents and information received from participants, we were able to identify 10 implementation strategies from the ERIC taxonomy [7] that are present in the implementation of COS-VF (see Table 1).

**Figure 2:**
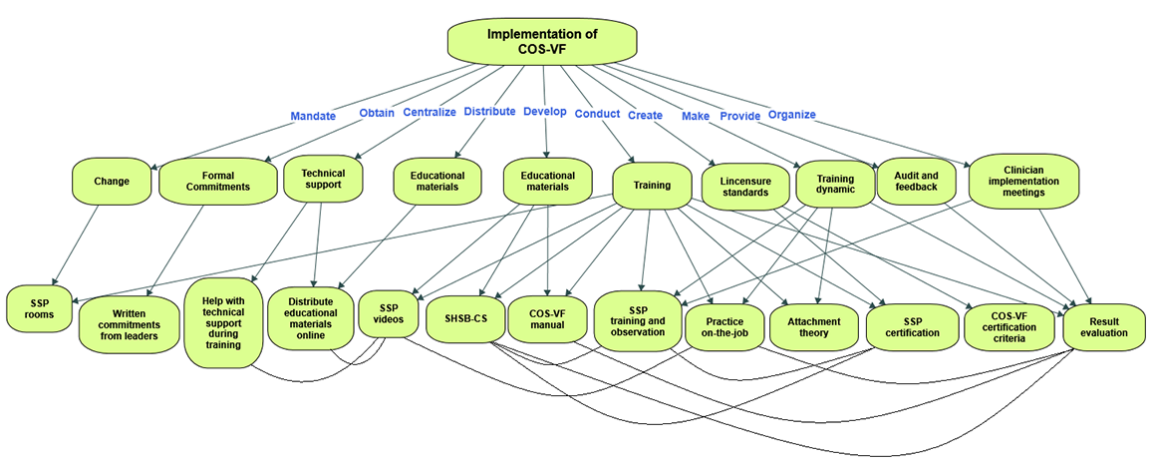
A concept diagram of the implementation strategies and their interrelationships. COS-VF: Circle of Security-Virginia Family; SBSH-CS: Secure Base-Safe Haven coding system; SSP: Strange Situation Procedure.

After implementation strategies were identified, named, and defined, they were organized according to Proctor and colleagues’ [5] seven dimensions (see Tables 2 and 3). The strategies and their interrelationships are depicted in Figure 2.

#### Develop Educational Materials

Before onset of therapist training in COS-VF, different educational materials were developed (ie, SBSH-CS and the COS-VF intervention manual) to train new therapists to a certain competency level and ensure intervention fidelity (see Table 2 and Figure 2). The use of manuals also facilitates replications by different researchers and increases comparability across studies using the same manuals [28]. Supervisors in Norway developed a Norwegian version of the COS-VF manual [29], which is the version currently used in Norwegian health and clinical services. This manual consists of six phases designed to guide therapists in (1) Assessment and treatment planning, (2) Establishing a supportive environment, (3), The didactics of the COS, (4) Building parents’ observation skills, (5) Increasing parental reflective functioning, and (6) Practice and integration. Manuals are distributed to trainees and form the basis for intervention delivery, fidelity, and certification criteria (see below).

#### Distribute Educational Materials

Clinicians in training receive access to all educational material via a cloud storage solution. This requires that trainees have a computer or laptop at their place of work that has access to the cloud service. Personnel at NIMH distribute educational materials online (eg, SSP training videos), provide technical support, and convey information concerning the training (see Table 3 and Figure 2).

**Table 1:** Implementation strategies identified in the Circle of Security-Virginia Family model.

Strategy	Definition
Develop educational materials	Develop and format manuals, toolkits, and other supporting materials in ways that make it easier for stakeholders to learn about the innovation and for clinicians to learn how to deliver the clinical innovation.
Distribute educational materials	Distribute educational materials (including guidelines, manuals, and supportive materials) in person, by mail, and electronically.
Conduct training	Plan for and conduct training in the clinical innovation in an ongoing way.
Make training dynamic	Vary information delivery methods to cater to different learning styles and work context, and shape the training in the innovation to be interactive.
Audit and feedback	Collect clinical performance data over a specific time period and give it to supervisors to evaluate and modify behavior.
Create or change credentialing and licensure standards	Create an organization that certifies clinicians in the innovation or encourage an existing organization to do so. Change governmental professional certification or licensure requirements to include delivering the innovation. Work to alter continuing education requirements to shape professional practice toward the innovation
Organize clinician implementation meetings	Develop and support teams of clinicians who are implementing the innovation, and give them protected time to reflect on the implementation effort, share lessons learned, and support one another’s learning
Obtain formal commitments	Obtain written commitments from key partners that state what they will do to implement the innovation.
Mandate change	Have leadership declare the priority of the innovation and their determination to have it implemented.
Centralize technical assistance	Develop and use centralized system to deliver technical assistance focused on implementation issues.

**Table 2:** Specifications of strategies used to implement the Circle of Security-Virginia Family model in health care services.

Strategy	Develop educational materials	Distribute educational materials	Conduct ongoing training	Make training dynamic	Audit and provide feedback
Actor(s)	Developers (United States) and supervisors	Developers (United States), supervisors, and technical support worker (NIMH^a^)	Supervisors	Developers (United States) and supervisors	Supervisors
Action	The developers developed a COS-VF^b^manual (Marvin and Whelan, 2010) and a Secure base-safe haven coding system (SBSH-CS, Marvin and Whelan, 2007) for the Strange Situation Procedure (SSP) that the therapists use during training and as part of the treatment after certification	Provide the clinicians with the information and materials they need to complete their COS-VF training, that is, the COS-VF manual, the SBSH-SC, as well as the practice SSP videos which are provided online	Training in attachment theory and observation of caregiver-child dyads based on the SSP (Ainsworth, 1968), the COS-VF manual, and measurements (ie, coding the SSP and COS interview)	The training provides knowledge through lectures on attachment theory and caregiver-child interaction, as well as real-life examples from SSP videotapes. Furthermore, the training allows the clinicians to practice their skills and get feedback from supervisors to enable them to attain the necessary competence	It is expected that the therapists complete the intervention with two caregiver-child dyads under supervision when working toward certification. The therapist should demonstrate appropriate skills in implementing all six phases of the intervention across the two cases
Target action	Therapists, clinicians in training	Clinicians in COS-VF training	Clinicians with a minimal of 3 years of college education within health and social sciences	Clinicians in training	Clinicians in training
Temporality	The COS-VF manual and the SBSH-CS were developed before the intervention was implemented	When they start section two of their educational course	Training starts before the intervention is implemented and lasts approximately 2.5 years	Ongoing	Audit and feedback begins when the clinicians start working with cases or families under supervision
Dose	The therapist and the clinicians in training use the manuals as part of the therapy with every case or family	They only receive educational materials once during their training	The educational course lasts for 10 (6-hour) days divided into two sections. The supervised clinical work starts after this section is completed. Altogether, the training period lasts about 2.5 years	The educational course lasts for 10 (6-hour) days divided into two sections. The supervised clinical work starts after this section is completed. Altogether, the training period lasts about 2.5 years	Clinicians are supervised every 3 to 4 weeks for about 1½ years. Thereafter, approximately every 6 to 8 weeks for the last half year of training
Implementation outcome(s) affected	Fidelity, sustainability	Fidelity, sustainability	Acceptability, appropriateness, fidelity, and sustainability	Fidelity	Fidelity
Justification	Manualized treatment makes it easier to train therapists to a certain level of competence, as well as ensuring fidelity to the intervention (Wilson, 1996)	The educational materials are given to all of the clinicians in training to make sure everyone has the materials they need, when they need it, which facilitates training	Research suggest that effective training consist of presenting information or knowledge, providing demonstrations either live or recorded, combined with practicing key skills in training setting (Joyce and Showers, 2002)	Training is more effective when the information delivery methods are varied to cater to different learning styles, and clinicians are able to practice their skills in work settings (Joyce and Showers, 2002)	Most skills can be introduced in the educational courses, but they need to practice at work with proper supervision to become successful therapists (ie, Fixsen and Blase, 2009; de Vries and Manfred, 2005; Joyce and Showers, 2002)

^a^NIMH: Network for Infant Mental Health.

^b^COS-VF: Circle of Security-Virginia Family.

**Table 3:** Specifications of strategies used in the implementation of the Circle of Security-Virginia Family model in health care services.

Strategy	Create or change credentialing and licensure standards	Organize clinician implementation meetings	Obtain formal commitments	Mandate change	Centralize technical assistance
Actor(s)	Developers (United States) and supervisors	Supervisors	Clinicians who take part in the COS-VF^a^model training	Providers (NIHM^b^)	NIMH’s technical support worker
Action	The therapist under training needs to be able to successfully code 80% of a set of 20 SSP^c^videos of caregiver-child dyads to become certified in coding attachment patterns using the SBSH-CS^d^. They also have to demonstrate competence while completing two cases under close supervision to become certified COS-VF therapists	Maintenance seminars are held at NIMH to provide the therapists an opportunity to discuss their experiences working with the COS-VF intervention and review videos of caregiver-child dyads to practice and maintain their skills	Clinicians have to obtain written commitments from their leaders that confirms that they are allowed to use 20% of their work hours on the COS-VF training	It is mandated that the clinicians in training have a SSP room available at their place of employment	Distributes educational information and materials online. Helps with technical support in issues related to COS-VF
Target action	Clinicians in training	Therapists	Leader(s) at the clinicians place of employment	Clinicians in training and their leaders	Therapists and clinicians in training
Temporality	Once during the educational course	One day each year	Before training	From the time they start working with case or families under supervision	Ongoing
Dose	There is no time frame for how long they have to complete the coding, or how many chances they get to succeed	6 hours	20% of their work hours on COS-VF training for the next 2.5 years	Ongoing	Ongoing or when needed
Implementation outcome(s) affected	Fidelity	Sustainability	Feasibility, penetration, and sustainability	Feasibility, appropriateness, and penetration	Acceptability, appropriateness, fidelity, and sustainability
Justification	This implementation strategy assures that the therapists have a certain competency level before they are allowed to treat patients and facilitates both fidelity to the intervention and evaluation of treatment results	Giving the therapist a chance to discuss their experiences and practice their skills facilitates fidelity and sustainability	Obtaining formal commitments ensure that both the clinicians who want to start COS-VF training and the leaders at their job are informed about what is expected of them and commit to doing so	The SSP room is a vital tool in evaluating the child’s attachment patterns, as well as an important part of the therapist assessment and treatment plan (Ainsworth, 1978)	Access to educational information and materials, as well as the technical support needed while in training makes it easier to complete the training and to implement the intervention

^a^COS-VF: Circle of Security-Virginia Family.

^b^NIMH: Network for Infant Mental Health.

^c^SSP: Strange Situation Procedure.

^d^SBSH-CS: Secure Base-Safe Haven coding system.

#### Conduct Ongoing Training

The target group for COS-VF training are infant mental health practitioners who work within child welfare services, foster care, and child mental health. Clinicians who apply for COS-VF training are obligated to have at least 3 years of college or university education within health and social sciences, supplementary clinical education, and clinical practice. The educational training contains two sections consisting of 5 days each, with certification criteria for each part: (1) the SBSH-CS and (2) the COS-VF intervention. Curriculum for section one is attachment theory and caregiver-child interaction, following 4 to 5 months of practicing coding videotapes of families in the SSP. The second section involves learning core elements and each step in the COS-VF intervention according to the manual. After these two sections are completed, clinicians start working under regular supervision on the job. The duration of training and supervision depends on the families the clinicians have in therapy (ie, on how long the therapist deems it necessary for the family to gain new understanding of how to meet and support their child’s needs according to attachment theory and change their parental behavior accordingly). It usually takes 2 to 2½ years from the start of training until clinicians become certified COS-VF therapists. The location of the training rotates between different regions of Norway for each new class to facilitate implementation of the intervention at the national level. This is part of the overall dissemination strategy where each strategy presented herein is implemented at each training site.

#### Make Training Dynamic

Training is made dynamic by using different methods of information delivery that cater to different learning styles by (1) Providing new skills through lectures, (2) Group work, (3) Watching videos of caregiver-child dyads with different attachment issues, as well as (4) Having to practice coding videos to a certain competence level before they can (5) Start working with families under supervision (see Table 2 and Figure 2).

#### Audit and Feedback

Clinicians in training must work on two families under supervision before they can become certified COS-VF therapists. Clinicians are divided into groups of four and receive supervision in a group setting either through Skype or face-to-face. Supervision is based on the principles of auditing and feedback where therapists and supervisors jointly watch and examine video recordings of therapists’ sessions with families. During supervision, there should be active reflection between the clinician and the supervisor, as well as effective engagement in a reflective dialogue concerning the clinician’s strengths and abilities around the circle when working with the caregivers. The underlying framework is the one of the “nested hands.” The supervisor is the “hands” to the therapist, the therapist the “hands” to the caregiver, and the caregiver the “hands” to the child (ie, the hands holding the child or caregiver or therapist in his or her experiences with casework or parenting around the circle).

In COS-VF, clinicians are supervised every 3 to 4 weeks for about 1½ years and then with reduced supervision approximately every 6 to 8 weeks for the last half year. During the first family intervention, supervisors have the primary role in implementing the intervention in cooperation with the clinicians in training, whereas the clinician takes the primary role with the second family. After certification, therapists no longer receive any formal supervision from the COS-VF providers (NIMH), but they can take part in maintenance seminars that NIMH organizes once a year (see Clinician Implementation Meetings below).

#### Create or Change Credentialing and Licensure Standards

COS-VF therapists under training must become certified in both (1) Coding attachment patterns of caregiver-child dyads using the SSP and (2) The intervention. As part of their training course, they learn to use the SBSH-CS and must successfully code 16 (ie, 80%) out of 20 SSP videos to become certified. They must pass the course before they can receive their certificate. Therapists in training are never told how many mistakes they had if they fail. They are simply given general feedback on whether they were close or far from passing to ensure the answers are never disclosed and increase the likelihood of passing the course by demonstrating competence rather than because of any nonspecific factors (eg, chance or poor course design).

To become certified COS-VF therapists, clinicians in training have to demonstrate competence in each of the following areas (each with a separate set of criteria): (1) assessment and treatment planning (eg, finding parents’ linchpins to choose appropriate video clips for treatment to demonstrate underutilized strengths), (2) therapist-parent interaction (eg, how the therapist is the “nested hands” for parents), and (c) therapist-supervisor interaction (eg, engaging in reflective dialogue with a supervisor). Certification is based on a joint reflective dialogue between the supervisor and therapist to allow judgments on whether the therapist is sufficiently competent to conduct the intervention without supervision.

#### Organize Clinician Implementation Meetings

Every year, the program providers (NIMH) invite COS-VF therapists to a maintenance seminar, where they discuss their experiences. These seminars allow the therapists to train and maintain their skills, as well as provides a forum for discussing their COS-VF-related experiences with other therapists. The health care services (ie, therapists’ workplace) must cover a small seminar fee, travel costs, overnight stays, and other expenses related to the seminar.

#### Obtain Formal Commitments

Clinicians who apply for COS-VF training must commit to using at least 10% to 20% of their work hours on COS-VF. To ensure this, they have to obtain a written consent by their immediate supervisor or leader at their workplace. This makes the likelihood of misunderstandings between leaders and their employees about the requirements of the training less probable, as well as making it easier for clinicians to spend the time they need on their training.

#### Mandate Change

To take part in COS-VF training, it is mandated that clinicians have access to an appropriate room for conducting the SSP (eg, video cameras and two-way mirror), or else they would not be able to carry out the intervention with families, which is part of the training. It also ensures intervention fidelity after certification, as SSP observations are part of the assessments that should be routinely conducted with each family before and after the intervention.

#### Centralize Technical Assistance

There are personnel at NIMH in charge of distributing all the information concerning COS-VF (ie, course information, educational materials, SSP training videos, etc), as well as technical support for clinicians in training (see Figure 2 and Table 3); an implementation strategy that makes sure they have easy access to everything they need throughout training, which makes it easier for them to succeed.

**Figure 3:**
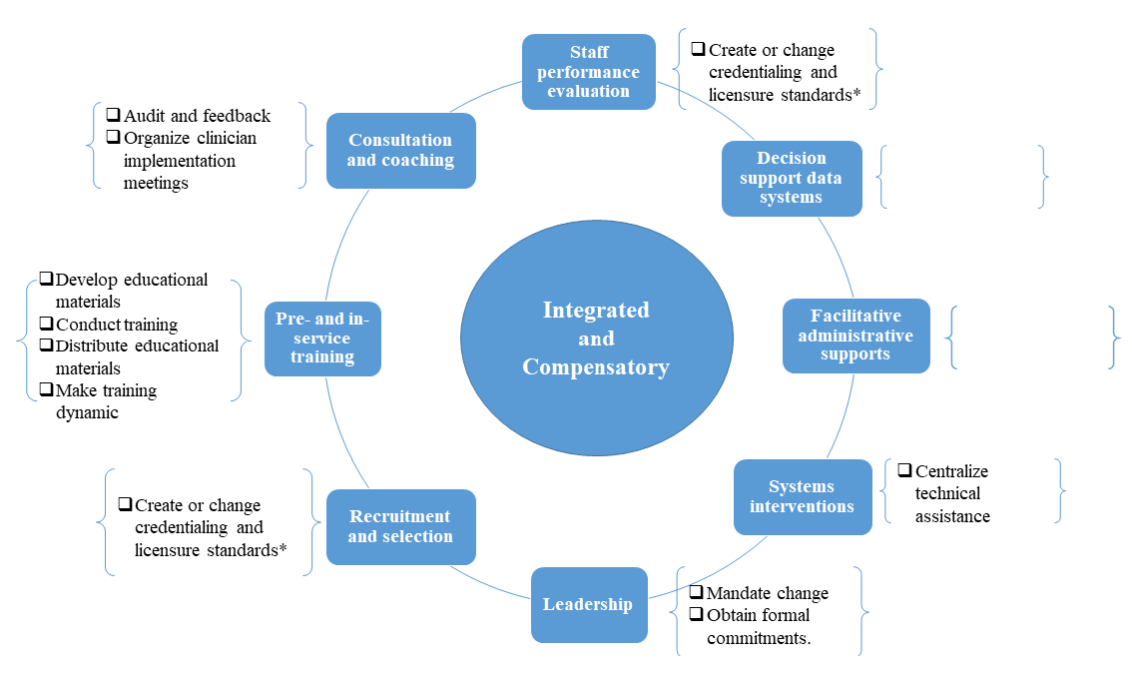
Display of the implementation strategies placed within the core implementation components. COS-VF: Circle of Security-Virginia Family. Asterisk (*): Create or change credentialing and licensure standards is mentioned twice in the digram as the strategy involves aspects which apply to both recruitment and staff selection and staff performance evaluation.

### Core Implementation Components

As the COS-VF providers were inspired by Fixsen and Blasé’s [14] core implementation components, we found it interesting to visualize which of the components were in place and which of the components needed more attention. This was done by organizing the strategies within their diagram of core implementation components. As depicted in Figure 3, pre- and in-service training has gotten a lot of focus in the implementation of COS-VF. There are also some strategies involved with systems interventions, recruitment and selection, consultation and coaching, as well as staff performance evaluation. We also added our own component and named it leadership, a component that has been taken into consideration during the implementation of COS-VF. Facilitative administration and decision support data system, however, do not have any strategies at this point.

## Discussion

### Principal Findings

The aim of this study was to provide a systematic and comprehensive review of strategies involved in the implementation of the COS-VF model in Norway. By combining informal interviews and documentation from COS-VF providers (ie, NIMH), 10 strategies were identified based on the ERIC taxonomy [7]: (1) develop educational materials, (2) distribute educational materials, (3) conduct ongoing training, (4) make training dynamic, (5) auditing and feedback, (6) create or change credentialing and licensure standards, (7) organize clinician implementation team meetings, (8) obtain formal commitments, (9) mandate change, and (10) centralize technical assistance.

Before COS-VF was implemented in Norway, different educational materials were developed and available (ie, SBSH-CS, SSP videos, and the COS-VF manual). Research suggests that manualized therapies make it easier to train therapists to a certain competency level, easier for supervisors to monitor trainees’ abilities, and facilitate future research. Previous research also suggests that manualized treatments increase intervention fidelity [28]. However, it is important to note that fidelity is more than just adherence to a set of well-defined procedures outlined in a manual but also includes competence in the delivery of an intervention and patient engagement, among other things [30]. Manuals solely provide a minimum operational description of how therapists are expected to behave and what they are expected to provide their patients [31]. Thus, it is important to consider who will be using these manuals, where, and what training is necessary for their effective use in practice.

When selecting and recruiting from applicants who want to take part in COS-VF training, there are several factors that need to be taken into consideration such as previous education, work experience, and current place of employment, that is, whether they are within the target group have access to families and mandate to conduct long-term therapy. However, it is still unknown what experience and credentials should be required when selecting staff for manual-based interventions to achieve effective use of treatment manuals [28], or which health care services have the organizational capacity to implement new practices [32]. Because staff and service selection are largely neglected areas within implementation research, it is difficult to give any clear directions regarding maintenance or improvement issues related to this area of the COS-VF implementation process [24]. Thus, it is crucial to conduct more research on staff and health care service selection, as these are important variables in promoting successful implementation.

Training in COS-VF is designed in a way that provides knowledge of theory, introduces components and rationale for key practice, provides opportunities to practice new skills, and receive feedback in a safe training environment (ie, ongoing, dynamic training, auditing, and feedback). This is consistent with research that learning a new intervention requires significant behavior change for therapists, as well as process guidance [33]; ie, close supervision, emotional support, result evaluation, and feedback based on practical experience. This is supported by Joyce and Showers’ [34] meta-analysis that shows that real learning and implementation occurs on the job, with supervision. Such on-the-job training, eventually, culminates in certification as COS-VF therapists (ie, created credentialing and licensure standards) and a level of competency, which suggests that graduates can deliver COS-VF without supervision. However, although training and supervision in COS-VF is well-attended to, it is an open question whether continued supervision should be provided after therapists finish their formal training to a greater degree than what is currently offered (ie, voluntary annual maintenance seminars). As Fixsen and colleagues [21] have pointed out, training and supervision are one of the principal ways in which behavior change is brought about not only at the start of the implementation but also throughout the lifespan of an intervention.

An implementation protocol can help implementation of an intervention, is useful, and contributes to the understanding of subsequent research, regardless of the intervention’s evidence base. Furthermore, one of the greatest advantages of developing an intervention protocol for COS-VF is that it facilitates transferability because the replication of an intervention is highly dependent on the conditions of the implementation (ie, whether or not the providers followed a protocol: [35]). Implementation fidelity will make it easier to replicate findings from research on the intervention when or if the implementation expands nationally and internationally, which again will ensure the generalizability of the results.

### Future Development

COS-VF therapists receive supervision during training; however, there is no evaluation of their work after they become certified. That is not to say that they do not receive evaluation by their employers; however, this is not a strategy that is part of the COS-VF implementation. The intention of staff evaluation is to assess the use and outcomes of skills therapists are taught in training and help them continue to improve their effectiveness with patients [21,36,37]. Evaluating therapist performance and using fidelity measures provides useful feedback concerning implementation efforts, training, and supervision. Furthermore, as previous research suggests that high fidelity implementation produces better outcomes for its recipients (ie, the patients; eg, [38]), this could be an area for quality development.

Previous research (ie, [21,39] ) proposes that frequent process and outcome reports guide decision making at the policy- and practice-level of organizations, as well as making it easier for organizations to continuously improve. However, there were no strategies involved in assessing key aspects of overall performance in COS-VF. This area should thus also receive more attention in further quality development of COS-VF. Furthermore, COS-VF has no strategies involved with administrative support; ie, components that give attention to policies, procedures, structures, culture, and climate, to assure alignment of these areas of an organization with the needs of the therapists. Previous research suggests that this is an important part of the implementation process that should not be disregarded [21,40] and could serve as an area for intermediary organizations such as the NIMH at the Regional Centre for Child and Adolescent Mental Health, to support health and clinical services.

### Limitations

A key limitation with this implementation protocol is that it was developed in a post-hoc manner, rather than during the planning stage of implementation. However, developing the implementation protocol as part of a reflective or evaluation phase allowed us to identify and critically appraise key aspects of the implementation process. This may point toward weaknesses in the implementation of COS-VF, which, in turn, makes it possible to identify areas in need of further improvement. In a sense, this may be one of the strengths of developing implementation protocols post-hoc and highlights why it is better to design such implementation protocols after the deployment of an intervention, rather than never. This implementation protocol shows that the competency area of the implementation of COS-VF is taken care of to a considerable extent, whereas the implementation of COS-VF in areas of organization and leadership is more limited and less developed.

Implementation protocols are constrained to local and contextual conditions under which a given intervention is implemented (even though this may be at a national or international level). Therefore, one should be careful in considering the potential usefulness of the outcomes of this study to other interventions or countries. However, although delimited to a specified intervention or context, implementation protocols may be the “missing link” necessary to replicate studies and to transfer theory and research into practice.

The third limitation involves the ERIC taxonomy [7] and the fact that the expert panel that participated in developing the taxonomy consisted mostly of implementation and clinical experts from the United States. It is possible that some strategies are more applicable in North-American settings and less applicable outside of North America. It may even be that there are unidentified strategies that are applicable outside American settings that are currently not included in the taxonomy. This could have affected the conceptualization of implementation strategies in this study. Nevertheless, to the best of our knowledge, there is no evidence that suggests that the compilation is not applicable across different contexts.

### Conclusions

This study describes the development of a post-hoc implementation protocol for the implementation of COS-VF in Norwegian health and clinical services. The development of the implementation protocol has made it possible to further develop and quality improve the implementation of COS-VF. Although COS-VF has yet to be evaluated, the identified implementation strategies may provide a valuable contribution to the understanding of subsequent research findings and blueprint for future implementation of COS-VF and, if possible, other interventions and in other countries as well. The implementation protocol will also make it easier for future research to replicate research findings and avoid “type III” errors.

## Abbreviations

AIF: Active Implementation Framework
COS-VF: Circle of Security-Virginia Family
ERIC: Expert Recommendations for Implementing Change
NIMH: National Network for Infant Mental Health
SBSH-CS: Secure Base-Safe Haven coding system
SSP: Strange Situation Procedure
